# Viral Strain Determines Disease Symptoms, Pathology, and Immune Response in Neonatal Rats with Lymphocytic Choriomeningitis Virus Infection

**DOI:** 10.3390/v11060552

**Published:** 2019-06-14

**Authors:** Jeffrey M. Plume, Dylan Todd, Daniel J. Bonthius

**Affiliations:** 1 Neuroscience Program, Carver College of Medicine, University of Iowa, Iowa City, IA 52242, USA; jeffrey-plume@outlook.com (J.M.P.); dylan-todd@uiowa.edu (D.T.); 2 Department of Pediatrics, Carver College of Medicine, University of Iowa, Iowa City, IA 52242, USA; 3 Department of Neurology, Carver College of Medicine, University of Iowa, Iowa City, IA 52242, USA

**Keywords:** cytokines, behavior, encephalomalacia, microcephaly, astrocytes, Bergman glia, olfaction, cerebellum

## Abstract

When infection with lymphocytic choriomeningitis (LCMV) occurs during pregnancy, the virus can infect the fetus and injure the fetal brain. However, type, location, and severity of neuropathology differ among cases. One possible explanation for this diversity is that fetuses are infected with different viral strains. Using a rat model of congenital LCMV infection, we investigated how differences in LCMV strain (E350, WE2.2, and Clone 13) affect outcome. Rat pups received intracranial inoculations on postnatal day 4. E350 initially targeted glial cells, while WE2.2 and Clone 13 targeted neurons. The E350 strain induced focal destructive lesions, while the other strains induced global microencephaly. E350 attracted large numbers of CD8+ lymphocytes early in the disease course, while Clone 13 attracted CD4+ lymphocytes, and the infiltration occurred late. The E350 and WE2.2 strains induced large increases in expression of pro-inflammatory cytokines, while Clone 13 did not. The animals infected with E350 and WE2.2 became ataxic and performed poorly on the negative geotaxis assay, while the Clone 13 animals had profound growth failure. Thus, in the developing brain, different LCMV strains have different patterns of infection, neuropathology, immune responses and disease symptoms. In humans, different outcomes from congenital LCMV may reflect infection with different strains.

## 1. Introduction

Lymphocytic choriomeningitis virus (LCMV) is a prevalent pathogen that infects large numbers of humans world-wide [1,2,3,4]. The viral infection is endemic in wild mice, which secrete the virus in large numbers. Humans can become infected when they contact fomites contaminated with the secretions of infected mice [5,6,7].

Symptoms of LCMV infections that are acquired postnatally are usually mild and consist of fever, malaise, and headache. In many cases, the infection is asymptomatic and never formally diagnosed [8].

A markedly different scenario unfolds when the infection occurs prenatally. LCMV can cross the placenta and infect the developing fetus, where the infection can induce severe and permanent injury [9]. LCMV is strongly neurotropic in the human fetus. As a result, the teratogenic effects of congenital LCMV infection are largely confined to the central nervous system, and especially to the brain and retina [10,11,12,13].

While LCMV infection can cause great damage to the human fetus, some are more severely impacted than others. The viral infection induces fetal demise in some, while others survive. Among those fetuses that do survive, LCMV induces severe brain injury in some cases but only moderate brain injury in others. Furthermore, the site of the neuropathology differs among cases. In some children with congenital LCMV infection, the injury is principally cortical or subcortical, while in others it is confined to the cerebellum or to the retinas. The form of neuropathology can also differ from case to case. The brains of some infected fetuses have prominent porencephalic cysts, suggesting a destructive process. Others have lissencephalic cortices, suggesting a neuronal migration disturbance, while still others have cerebellar hypoplasia, suggesting impaired neurogenesis [10]. 

As a result of the divergent sites, severity, and forms of neuropathology, children with congenital LCMV infection have a wide variety of outcomes. While those with microencephaly and periventricular calcifications are always severely cognitively impaired and have spastic quadriplegia, children in which the LCMV infection induces isolated cerebellar hypoplasia are ataxic, but have only mild cognitive impairment. For children in whom the pathology is confined to chorioretinitis, LCMV is principally a blinding disease [14]. 

Why prenatal infection with LCMV induces such a wide variety of pathology and outcomes is unknown. Part of the reason appears to be due to differences in the gestational timing of infection, since the cellular targets of infection within the brain and the immune response to the virus change over the course of development [15].

Another possibility is that the fetuses are infected with different *strains* of the virus. Dozens of LCMV strains have been isolated from infected humans, experimental animal models, and natural rodent reservoirs [16]. Studies have shown that infection with these different strains can induce substantially different biological effects in several model systems. For example, in rhesus macaques, the WE-54 strain of LCMV causes a fatal hepatitis, while the Armstrong strain, which shares 88% amino acid homology with WE-54, causes no disease in macaques [17,18]. Similarly, in adult mice, depending on their affinity to bind alpha-dystroglycan, some strains of LCMV will infiltrate the splenic white pulp, ablate the cytotoxic T-lymphocyte response, and establish persistent infection, while other genetically closely related strains will not [19]. However, to date, the importance of viral strain has not been examined in congenital LCMV infection.

During the course of brain development, all mammalian species pass through a period of rapid brain growth, referred to as the “brain growth spurt.” During this period, the developing brain is particularly vulnerable to teratogenic agents, including chemicals and viruses [20]. While all mammalian species experience a brain growth spurt, the timing of this event, relative to birth, varies among the species. In humans, the brain growth spurt begins in the second trimester and peaks in the third trimester. In contrast, in rats, the brain growth spurt begins shortly after birth and peaks during the second postnatal week [21,22]. Therefore, to mimic human prenatal exposures during the brain growth spurt, rats must be exposed during the early *postnatal* period. For this reason, the neonatal rat is commonly used as a model system for the study of human prenatal exposures [23,24]. 

The neonatal rat inoculated with LCMV provides an excellent model system of human congenital infection [25,26]. Because the rat brain is immature at the time of birth, relative to the human’s, early postnatal infection with LCMV in the rat mimics human prenatal infection [22]. Inoculation of the neonatal rat with LCMV can faithfully recapitulate virtually all of the neuropathologic changes observed in children with congenital LCMV infection [15,27]. All published research to date using the rat model of congenital LCMV has focused on a single strain of LCMV, which is Armstrong-4 [10,15,25,28,29,30].

The present study uses the rat model to examine the importance of strain in the effects of LCMV in the developing brain. The three strains of LCMV compared were E350, Clone 13, and WE2.2.

The E350 strain was cloned from the original LCMV strain isolated by Armstrong and Lillie in 1933. This strain is characterized by the intense lymphocytic infiltration induced in the meninges and eyes of infected experimental mice and monkeys [31]. The Clone 13 strain is similar to the E350 strain, differing by only a few amino acids. However, the Clone 13 strain contains a single point mutation in the viral polymerase, which underlies its capacity to persist, as well as an F260L mutation in its glycoprotein, which influences its receptor binding affinity. Studies in adult mice have shown that, as a result of these mutations, Clone 13 has enhanced intracellular replication and binds strongly to dendritic cells, resulting in immunosuppression and persistent infection [19,32,33]. The WE2.2 strain is phylogenetically distant from the Clone 13 and E350 strains with many more mutations. It is non-immunosuppressant and uses a different receptor than Clone 13 [19,34]. In addition, it induces different biological effects than E350 in adult animals. E350 destroys the liver cells of adult mice, but does not kill guinea pigs. In contrast, WE2.2 causes no significant liver injury in adult mice, but is highly lethal to guinea pigs [35]. Thus, E350, WE2.2, and Clone 13 were chosen for comparison in this study because they differ in their receptor binding affinities, effects on the immune response, and overall virulence. 

This research examined the importance of viral strain in determining the effects of LCMV on the developing brain in a rat model. Our findings demonstrate that different strains of LCMV induce different disease symptoms, patterns of infection, forms and severity of pathology, and immune responses.

## 2. Materials and Methods

### 2.1. Animals

The experiments were performed with Lewis Rats (Harlan Sprague-Dawley), housed in biosafety level-II containment quarters at the University of Iowa Animal Care Facility. Timed-pregnant rats were observed every 24 h to establish the time of birth of the litters. The day of birth was defined as postnatal day zero (PD0). Animals were monitored daily for signs of morbidity and mortality. Body weights of individual pups were recorded every other day. All experiments were conducted with the approval of the Institutional Animal Care and Use Committee at the University of Iowa (Protocol approval #1107147; date 31 August 2011).

### 2.2. Virus

All LCMV strains used in these studies (E350, Clone 13, and WE2.2) were generously donated by Dr. Juan de la Torre (Scripps Institute, San Diego, CA, USA). Each viral strain was expanded on a semi-confluent monolayer of baby hamster kidney (BHK) cells. After 3 days, the supernatant was collected, and viral titer was determined by plaque assay.

### 2.3. Infections

Animals were injected in the right cerebral hemisphere on PD4 with 1000 plaque forming units (PFU) of LCMV in a volume of 5 µL of Dulbecco’s Modified Eagle Medium (DMEM) via a Hamilton syringe with a needle depth guard. Uninfected control littermates received sham intracerebral injections of 5 µL of DMEM. Uninfected animals were fostered to unexposed mothers with unexposed litters.

### 2.4. Perfusions and Pathological Analysis

At a series of ages post-infection (PD8, 10, 14, 18, and 22; *n* = 6–8 at each age), the rat pups were anesthetized with ketamine/xylazine and perfused via the left cardiac ventricle with saline, followed by 4% paraformaldehyde in 0.1 M phosphate buffer. Brains were harvested and stored for 24 h in 4% paraformaldehyde fixative. Cerebellums and olfactory bulbs were isolated.

For half of the animals (*n* = 3–4 per treatment and time point), the tissues were prepared for frozen sectioning. These tissues were placed in 30% sucrose in 0.1 M phosphate buffer at 4 °C until they sank. Forty µm-thick sections of these tissues were cut with a freezing microtome. The olfactory bulbs were sectioned horizontally, and the cerebella was sectioned sagittally. All sections were saved, and a 1:10 series of sections were mounted on slides and Nissl stained with cresyl violet for detection of pathological changes. The other sections were stored at −20 °C in cryoprotectant for immunohistochemistry.

The cerebellums from the remaining animals (*n* = 3–4 per treatment and time point) were prepared for paraffin embedment. These tissues were dehydrated through a graded series of alcohol, then embedded in paraffin. Four µm-thick sections were cut in the midsagittal plane with a rotary microtome. The sections were mounted on slides and stained with hematoxyline and eosin (H&E).

### 2.5. Quantifying Cross-Sectional Area of the Cerebellum

In each series of 40 µm-thick cerebellar sections, the mid-sagittal section of the cerebellar vermis was identified as that section in which lobules I and X are in closest approximation and in which no deep cerebellar nuclei are present. In this specific section from each animal, the outer perimeter of the tissue was traced with the freehand drawing tool in ImageJ (NIH) (Bethesda, MD, USA), and the area of the section was quantified using the measure macro.

### 2.6. Immunohistochemistry

Immunohistochemical staining for LCMV and glial fibrillaryacidic protein (GFAP) antigens were performed on floating sections. Tissue sections were washed three times with phosphate-buffered saline (PBS) and incubated with a blocking solution of 2% goat serum plus 0.1% Triton-X for one hour. Incubation with the LCMV (Guinea Pig Polyclonal, Dr. Juan De la Torre, Scripps Institute, San Diego, CA, USA) and GFAP (Rabbit Polyclonal, Millipore Sigma (Burlington, MA, USA), G9269) primary antibodies was performed at 1:500 and 1:1000 dilutions, respectively, in blocking solution for at least one hour at room temperature or overnight at 4 °C with continual rocking. Samples were washed three times with PBS and exposed to a cocktail containing anti-Guinea pig AlexaFluor^®^ 488 (Life Technologies™) (Waltham, MA, USA) and anti-mouse AlexaFluor^®^ 568 (Life Technologies™) secondary antibodies diluted to 1:200 in blocking solution for one hour at room temperature and then washed three times with PBS. Samples were mounted onto microscope slides and fixed with mounting media containing DAPI (Vector^®^ Labs) (Burlingame, CA, USA).

### 2.7. Negative Geotaxis Assay

**Figure 1:**
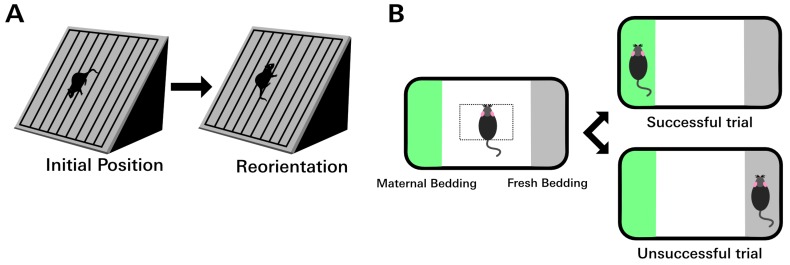
Behavior tests assessing motor and olfactory function. (**A**) In the rodent negative geotaxis assay, the animal is placed facing downhill on an incline. After PD7, young rodents will reflexively reorient to face uphill. The amount of time required to complete the 180° turn can be used to quantify a gross motor deficit. Failure was defined as taking longer than 60 s to complete the task. (**B**) In the olfaction discrimination assay, the animal is placed in the center of a rectangular area to either side of which is either maternal (home cage) bedding or fresh bedding. Between PD12 and PD14, rat pups have a strong preference for maternal bedding. Animals with olfactory deficits are slower to cross to the maternal side or may fail completely by choosing the fresh bedding.

Some of the animals used for the histopathologic analyses described above also underwent behavioral testing (*n* = 6–7 per treatment group). The negative geotaxis asssay is a test of motor function that exploits a pup’s natural tendency to orient its body uphill on an inclined plane (Figure 1A) [36]. In rats, this behavior is observed as early as PD7 and requires no conditioning trials [37]. PD10 animals were placed facing downhill on a 30° slope, and the time required to make a 180° turn was recorded. Inability to complete the task within 60 s was recorded as a failure. The inclined surface was coated with a wax barrier, which was replaced between trials to prevent possible scent distractions. Additionally, animals were rotated each trial such that no two animals from the same cage were tested consecutively. The experiment was conducted in quadruplicate for each animal.

### 2.8. Olfaction Discrimination Assay

Nest seeking behavior, in which pups seek maternal bedding, is a natural and adaptive response. Isolating a pup from its nest removes it from its food source, shelter, protection, and social interactions. Therefore, nest seeking is strongly reinforced and necessary for survival. The olfaction discrimination assay utilizes nest seeking behavior to test olfactory function in rat pups. The test relies on the natural tendency of a rat pup to move toward the familiar odor of home cage bedding [38].

PD12 animals were placed in a 30″ × 20″ × 8″ plastic bin filled with maternal (home cage) bedding on one side and fresh (unfamiliar) bedding on the other, separated by a region that contained no bedding (Figure 1B). A 3 cm^2^ square was demarcated in the center of the cage. Animals were placed in the center of the demarcated region and allowed to move freely. The choice of bedding was recorded over four trials. Choice of bedding was determined by the placement of a front paw on a line that demarcated the bedding area. Directional placement of the animals at the beginning of each trial was altered randomly between trials to control for right or left directional preference. Trials in which the animal chose the fresh bedding instead of maternal bedding were recorded as failures. The number of animals per group that failed at least one trial and the average number of failures per animal per group were calculated.

### 2.9. Isolating Leukocytes from Brain Tissue

A second group of animals was used to compare immune cellular responses induced by infection with the different strains. Following intracerebral inoculation with one of the viral strains or with DMEM on PD4, brains were harvested on PD14 or PD22 (*n* = 4–6 per treatment and per time point). The brains were weighed, then mechanically dissociated with 25-gauge needles in digestion buffer (Hank’s balanced salt solution HBSS with Ca^2+^, Mg^2+^, 1 mg/mL collagenase D (Roche©) (Indianapolis, IN, USA) and 100 µg/mL DNAse (Roche©) with 25 mM HEPES with 2% FBS). The tissue was further dissociated by placing the homogenate in a petri dish and dissecting with a razor blade. Each brain was diluted in 7 mL of digestion buffer and chemically digested at 37 ° for 30 min with continuous rocking. During the chemical digestion period, the samples were periodically pipetted through a 1000 µL pipette tip to further dissociate the tissue. To terminate the digestion, 70 µL of EDTA was added for 5 min at room temperature, and the mixture was placed in an ice bath for 5 min. Homogenates were filtered through a 70 µm filter, washed with RPMI plus 2% FCS, and spun at 1300 rpm for 8 min. The supernatant was discarded, and the resulting filtrate was suspended in 30% percoll (GE Healthcare©) (Chicago, IL, USA) plus RP10 (RPMI 16–40, Pen/Strep, L-glutamine, 10% Fetal Calf Serum). The percoll solution was spun at 1300 rpm for 20 min, producing a lymphocyte-rich pellet. The supernatant was discarded, and cells were washed twice with RPMI plus 2% FCS and diluted in RP10.

The number of live cells was determined by trypan blue exclusion. Lymphocytes were diluted to a final concentration of 1 × 10^6^ cells per 100 µL. The lymphocytes were pelleted at 1300 rpm for 8 min, then subjected to Fc receptor blockade in 50 µL of 4% goat serum in PBS for 15 min at 4 °C. Cells were then incubated with an antibody cocktail diluted in FACs buffer for 15 min. Antibodies included: 1:200 dilution of FITC anti-rat CD45 (BioLegend^®^, San Diego, CA, USA), 1:100 dilution of PE anti-rat CD8a (BioLegend^®^, San Diego, CA, USA), and 1:100 of dilution PE/Cy7 anti-rat CD4 (BioLegend^®^, San Diego, CA, USA). After the incubation, 100 µL of FACs buffer was added to the existing cocktails, and the solution was spun down at 1100 rpm for 5 min. Cells were washed twice with 2 mL PBS and then fixed with 4% PFA for 15 min. The cells were washed twice with 2 mL of PBS, then resuspended in 200 µL of FACS buffer for multicolor flow cytometry analysis with the BD Accuri™ (San Jose, CA, USA) c6 flow cytometer. Lymphocyte populations were determined by forward and side scatter signal.

The percentage of Hematopoietic cells was determined after gating for lymphocytes based on FL1 signal. The percentage of CD8+ and CD4+ cells was determined within the CD45+ population after compensating for fluorescent spillover. The absolute number of each cell type per brain was determined by multiplying the number of cells per microliter, calculated by the flow cytometer, by the total volume of diluent per brain sample prior to flow analysis. Values are presented relative to total brain weight.

### 2.10. Gene Expression Analysis

A third set of animals was used to compare cytokine gene expression patterns induced by infection with the different strains. Rat pups were injected intracerebrally on PD4 with DMEM or one of the LCMV strains, as described above. On PD8, PD14, or PD22 olfactory bulbs and cerebella were dissected and isolated from each brain (*n* = 6–7 per treatment and time point). Tissues were flash frozen in liquid nitrogen and stored at −80 °C until further use. RNA was isolated with the TRIzol RNA Isolation Reagent (Invitrogen™) (Carlsbad, CA, USA) according to the manufacturer’s protocol. Two µg of RNA was reverse transcribed with Superscript-II^®^ (Life Technologies™), and the resulting cDNA was diluted to 300 µL in nuclease-free water. Five µL of the resulting solution were used as a template for subsequent reactions. Gene expression of Interferon-Gamma, Interleukin-1 Beta, and Tumor Necrosis Factor Alpha were measured by TaqMan^®^ Probe-Based Gene Analysis (Applied Biosystems^®^) (Beverly, MA, USA). Real-time reactions were performed on the 7900 RT PCR system (Life Technologies™) in a 20 µL volume at the University of Iowa Genomics Division. TaqMan^®^ Ribosomal RNA Control Reagent (Applied Biosystems^®^) expression was measured as an internal control for each condition. Expression was quantified relative to similar tissues from uninfected controls using the 2^−ΔΔCT^ method.

### 2.11. Statistical Analyses

Body weights and brain weights were analyzed by repeated measures analysis of variance (ANOVA). Spleen weights and cerebellar cross section areas were analyzed by one-way ANOVA. To determine the extent to which spleen weights and brain weights were affected, relative to overall body weights, the ratios of spleen weight-to-body weight and brain weight-to-body weight were calculated and analyzed by ANOVA. For the behavioral studies, righting times, and average number of failures on the negative geotaxis assay were analyzed by one-way ANOVA, while the percentage of animals that failed for each treatment group were analyzed by Chi-square analysis. For the olfaction discrimination test, the average number of failures per treatment group were analyzed by one-way ANOVA, and the percentage of animals that failed at least once for each group were analyzed by Chi-square analysis. Lymphocyte identities and cytokine levels were analyzed by MANOVA. All analyses were conducted using SPSS statistical software (IBM Corp. Released 2013. IBM SPSS Statistics for Windows, Version 22.0. Armonk, NY: IBM Corp.). Consequently, *p*-values less than 0.05 were deemed significant. Post-hoc analyses were conducted using Bonferroni’s adjustments for multiple comparisons.

## 3. Results

### 3.1. Different LCMV Strains Produced Different Diseases

**Figure 2:**
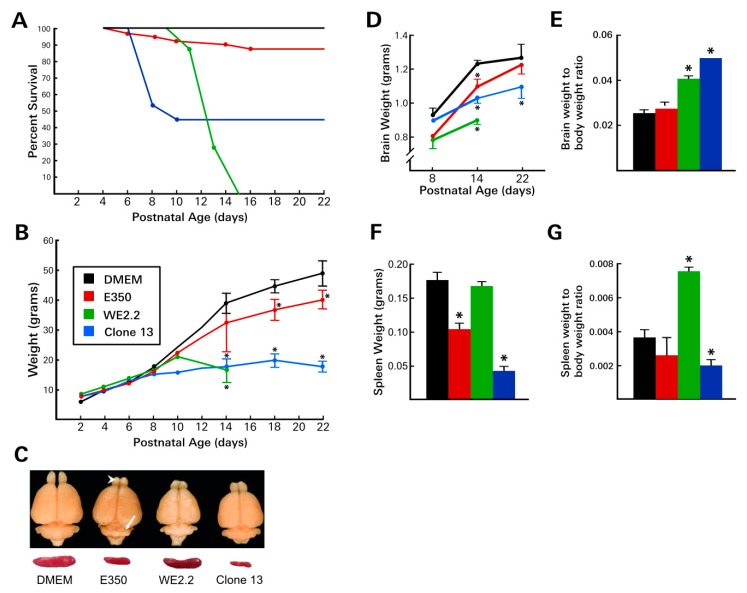
The different LCMV strains induced different diseases. (**A**) Following intracerebral inoculation on PD4, the various strains of LCMV induced markedly different patterns of mortality. The WE2.2 strain caused mortality starting on PD10, and all infected animals were dead by PD15. In contrast, the E350 strain caused very little mortality, and none after PD16. Clone 13 caused high rates of mortality during PD6-PD10, but no mortality at later time points. (**B**) Body weight deficits differed among the strains. The E350 strain caused only mild growth deficits. In contrast, Clone 13 and WE2.2 induced profound growth failure. (**C**) Representative brains and spleens at PD14 from each treatment group. All strains induced microencephaly, but the severity differed among strains. Focal destructive lesions in the olfactory bulb (arrowhead) and cerebellum (arrow) occurred only with the E350 strain. E350 and Clone 13 reduced spleen size, while WE2.2 did not. (**D**) Brain weights were reduced by all three strains, but most substantially by WE2.2. (**E**) Despite the microencephaly, brain weight-to-body weight ratios were increased in animals infected with WE2.2 and Clone 13 because of the greater impairment of body growth, compared to brain growth. (**F**) Spleen weights were substantially reduced by infection with E350 and Clone 13, but not by WE2.2. (**G**) Ratios of spleen weight to body weight were differentially affected by the viral strains due to different degrees of microsplenia and body growth. All data points represent means and error bars represent standard error of the mean. * Significantly different from uninfected control (Dulbecco’s Modified Eagle Medium (DMEM) injected animals.

Following inoculation with the E350 strain, nearly all of the rat pups survived (Figure 2). At PD 22, when the experiment concluded, 86% of the E350 pups remained alive. These animals were well-groomed, healthy-appearing, active, and exhibited no discernible evidence of systemic disease, except for a marked ataxia, as described below. A modest decrease in weight gain was observed in the E350 infected pups. They weighed approximately 10% less than DMEM controls from PD10 through PD 22.

In marked contrast to the E350 animals, the pups infected with the WE2.2 strain had poor survival and exhibited frank growth failure. Following intracranial inoculation on PD4, the WE2.2 infected animals began to show evidence of disease around PD 10. Symptoms included anorexia and ruffled fur—effects not observed in the E350-infected pups. Similar to the E350 animals, the WE2.2 infected animals became ataxic at PD 10. However, in contradistinction to the E350 pups, they also developed frequent convulsions. All animals infected with the WE2.2 strain failed to gain weight after PD 10 and invariably died 9–11 days post-infection. For most animals, death was preceded by severe anorexia and convulsive seizures, which were signs of disease that were never present in any of the E350 animals.

Infection with the Clone 13 strain produced a third pattern of disease. By 4 days post-infection, virtually all of the animals developed an unhealthy appearance, characterized by poor grooming, ruffled fur, and anorexia. During this acute illness, many of the animals lost weight. The effects of the stunted growth were persistent and dramatic; none of the infected animals recovered from the early weight loss. By PD 22, the pups infected with Clone 13 weighed less than half their littermate controls. The illness induced by Clone 13 peaked between PD 8 and PD 12, at which time it produced a high mortality. 65% of the Clone 13 animals died during this time frame. However, if the animals survived that period of acute illness, then their prospects for continued survival were excellent. No further deaths occurred among the Clone 13 animals after PD 12. The Clone 13 animals did not develop overt neurological symptoms. Unlike the E350 animals, they did not develop ataxia, and unlike the WE2.2 animals, they did not develop convulsions.

Thus, the three strains of LCMV produced markedly different disease states in neonatal rats. While each of the viral strains induced symptoms, those symptoms were unique to each strain and differed in their mortality, somatic growth disturbance, grooming, and neurological symptoms.

### 3.2. Different LCMV Strains Induced Different Patterns of Growth Disturbance and Pathology in Spleen and Brain

Some, but not all, LCMV strains impaired spleen growth. The WE2.2 strain, which substantially impaired somatic body growth generally, caused no reduction in spleen size. In contrast, the E350 strain, despite causing little impairment with somatic growth, markedly reduced spleen size. In further contrast, the Clone 13 strain induced an even more severe reduction in spleen size than did the E350 strain. To assess the effect on spleen size relative to body size, the ratio of spleen weight to body weight was calculated for each treatment. As shown in Figure 2, the ratio was significantly higher than control in the WE2.2 group and significantly lower in the Clone 13 treated animals. This reflected the fact that spleen size was relatively spared, relative to body size, in the animals treated with WE2.2, while, in contrast, the spleen was particularly affected, relative to body size, in the animals infected with Clone 13. Thus, the LCMV strains induced different degrees of spleen size reduction, and these differences in spleen growth were not simply a consequence of reduced body size.

**Figure 3:**
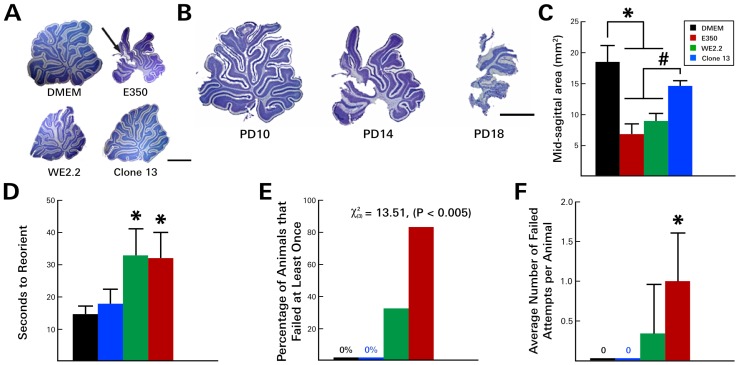
The different LCMV strains induced different cerebellar pathology and degrees of motor deficits. (**A**) Nissl-stained, 40-micron-thick sections through the midsagittal cerebellum on PD14. All three viral strains visibly reduced cerebellar size. However, only E350 induced destructive lesions (arrow). (**B**) The destruction of the cerebellum by E350 began at the tips of the dorsal lobules on PD10, then progressed to virtual total obliteration of the cerebellum by PD18. (**C**) Midsagittal areas of the cerebellum. E350 and WE2.2, but not Clone 13, significantly reduced cerebellar cross sectional area. In the rodent negative geotaxis assay, rat pups infected with E350 and WE2.2, but not Clone 13, took longer to reorient (**D**), had greater percentages of animals that failed (**E**), and had a greater number of failures per attempt (**F**). * Significantly different from DMEM (*p* < 0.05). # Significantly different from Clone 13 (*p* < 0.05). Scale bars represent 2 mm.

**Figure 4:**
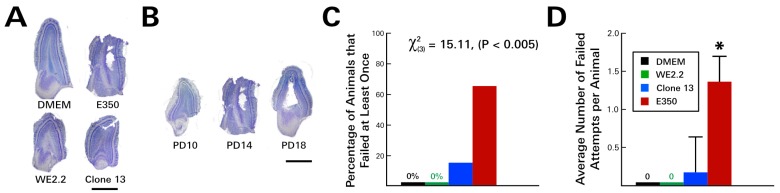
The different LCMV strains induced different types of olfactory bulb pathology and different severity of olfactory discrimination behavioral deficits. (**A**) Nissl-stained 40-micron-thick horizontal sections through the olfactory bulb on PD14. All three viral strains visibly reduced olfactory bulb size. In addition, the E350 strain induced encephalomalacia within the olfactory bulb. (**B**) In the olfactory bulb of rats infected with the E350 strain, destruction began within the subependymal zone on about PD 14 and progressed outward, producing a large porencephalic cyst at the core of the olfactory bulb by PD18. The severe damage to the olfactory bulb in E350 rats produced a marked deficit in the olfaction discrimination assay, so that E350-infected animals had a higher percentage of failing animals (**C**) and a higher average number of failed attempts per animal (**D**) than did groups infected with any other strain. * = significantly different from all other groups (*p* < 0.05). Mag bars represent 1 mm in A and B.

Each of the strains also impaired brain size and brain tissue integrity, and the strains again differed in their patterns and effects. Infection with the E350 strain caused only a modest reduction in brain size overall. However, this strain induced progressive destructive lesions within the cerebellum (Figure 3) and olfactory bulb (Figure 4). Beginning about 6 days post-infection (~PD10), pathological lesions began to form in the dorsal cerebellar lobules. Over the next 10 days, these destructive lesions progressed, eventually involving all lobules and leaving the cerebellum virtually obliterated. These pathologic changes in the cerebellum were accompanied by a marked ataxia in which the animals exhibited difficulty walking and righting themselves from a supine position. Over this same time period, the E350 virus induced similar encephalomalacia within the olfactory bulbs. In the olfactory bulbs, the destructive lesions began within the subependymal zone and progressed outward, producing a porencephalic cyst at the core of the olfactory bulb. 

Infection with Clone 13 induced a very different set of neuropathologic effects. Clone 13 induced moderate overall microencephaly, which was greater in its magnitude than that observed with E350. However, unlike E350, Clone 13 did not induce destructive lesions within the cerebellum, olfactory bulbs, or elsewhere. Instead, in both the cerebellum and olfactory bulbs, Clone 13 induced hypoplasia, leaving those structures markedly smaller than controls, but histologically intact.

The WE2.2 strain led to the most severe overall microencephaly, reducing brain weight by 28%, relative to uninfected controls, at PD 14. The microencephaly was significantly more severe at PD 14 than that induced by either of the other viruses. However, unlike E350, the growth failure in the brain was global, and no regions were more affected than others. Both the cerebellum and the olfactory bulb were hypoplastic, but histologically intact, and had no evidence of destructive lesions. Brain weight to body weight ratios revealed significant differences among the treatment groups. Despite the fact that the brains of all of the infected animals were reduced in size, their weight, relative to body weight, was increased. The brain-to-body ratios were significantly increased in the WE2.2 and Clone 13 groups. This increased ratio reflected the fact that body growth was severely impaired in the WE2.2 and Clone 13 groups, to a greater extent than was brain growth.

### 3.3. Different LCMV Strains Induced Different Behavioral Deficits

As noted above, following inoculation with the E350 strain on PD 4, the animals developed cerebellar destructive lesions and became acutely ataxic by PD 10. These changes led to poor performance on the negative geotaxis assay (Figure 3). On PD 10, the E350-infected animals required an average of 33 s to complete the task, compared to only 14 s for the uninfected control animals. Additionally, many of these animals could not complete the task—a deficit that was unique to the E350 and WE2.2 animals. At PD 10, 83.3% (5/6) of the E350 infected animals failed the task at least once, with each animal failing, on average, one out of the four attempts. By PD 14, the test was discontinued because nearly all of the E350-infected animals were failing to complete the task in less than 60 s, and their instability on the platform raised concerns regarding animal safety.

The WE2.2-infected animals also became ataxic and performed poorly on the negative geotaxis assay. In marked contrast, the animals infected with the Clone 13 strain did not become ataxic, and they performed well on the negative geotaxis assay. These animals performed similarly to controls, completing the task in 18 s, on average, with no failures by any of the animals across any of the four trials.

At the same time that the E350-infected pups developed cerebellar lesions, they also developed olfactory bulb destruction. As a result, when these animals underwent an olfaction discrimination task on PD 12, they performed significantly worse than uninfected controls (Figure 4). Unlike uninfected control animals, which all chose bedding from their home cage over fresh bedding on all trials, the E350 animals failed the task on many occasions. Specifically, 66.6% (4/6) of the E350 infected animals failed the task at least once, with each animal choosing fresh bedding, on average, 1.3 times over the course of four trials.

In contrast to the E350-infected animals, the pups infected with the WE2.2 and Clone 13 strains did not develop olfactory bulb encephalomalacia and correspondingly performed well on the olfactory bulb discrimination test (Figure 4). The WE2.2 pups had no failures over any of the trials, and the Clone 13 pups had only a single animal fail the olfaction discrimination task on one of four trials. Thus, the various strains of LCMV induced different behavioral deficits, which closely reflected differences in neuropathology.

### 3.4. Different LCMV Strains Induced Different Forms and Degree of Neuropathology within the Cerebellum

Previous studies utilizing the Armstrong-4 strain in the neonatal rat have demonstrated that disruption of Bergman glia structure is an important component of the pathologic changes that occur within the developing cerebellum. Bergman glia are GFAP-expressing cells whose radial orientation within the molecular layer of the cerebellum plays a key role in guiding granule cells to their mature locations. The Armstrong-4 strain of LCMV targets Bergman glia and alters their configuration.

**Figure 5:**
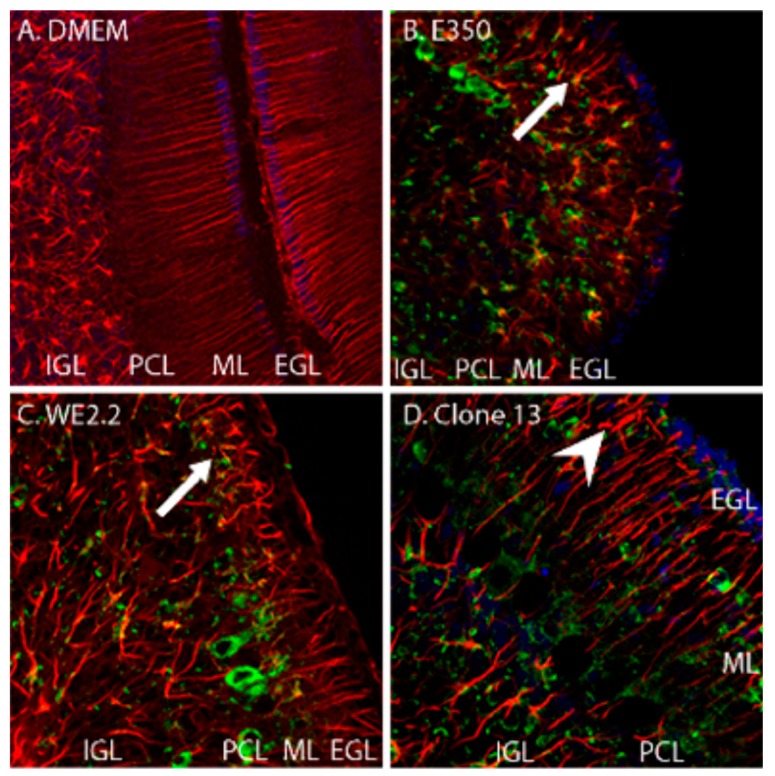
The different strains of LCMV induced different forms and severity of Bergman glial pathology. Pictured here are immunohistochemically stained (GFAP: red, LCMV: green, DAPI/nucleated cells: blue) of PD14 cerebellar tissue. (**A**) In control (uninfected) animals, Bergman glia are GFAP-positive cells that have an orderly, parallel, and unbranched pattern extending radially through the molecular layer. (**B**) In the animals infected with E350, the Bergman glia structure is radically altered (arrow). The cells are substantially foreshortened, branch abnormally, and have lost their parallel arrangement. (**C**) In animals infected with WE2.2, Bergman glia structure is also substantially altered (arrow), but less severely than that observed with E350. (**D**) In the animals infected with Clone 13, the Bergman glia were far less pathologically affected. The Bergman glia exhibited some abnormal branching (arrowhead) and deviation from their normally straight shape, but the length of their processes and their parallel configuration were generally preserved. IGL = Internal granule cell layer; PCL = Purkinje cell layer; ML = Molecular layer; EGL = External granule cell layer. Magnification is 200× for A, B, C, and D.

As shown in Figure 5, the E350, WE2.2, and Clone 13 strains all altered the normal structure and arrangement of Bergman glia. However, the severity of the pathologic changes differed among the strains. The E350 strain radically altered Bergman glia. In the E350 infected pups, the Bergman glia processes were greatly foreshortened and exhibited abnormal branching, and the cells completely lost their parallel configuration. The WE2.2 strain caused similar, but slightly less severe, changes to the Bergman glia. In contrast, the Clone 13 strain induced only minor changes in the Bergman glia. Those changes included some branching in the otherwise unbranched structure and some “corkscrewing” instead of the usually straight shape. However, the Bergmann glia were not foreshortened and the cells maintained their basic parallel relationships in the Clone 13 animals.

**Figure 6:**
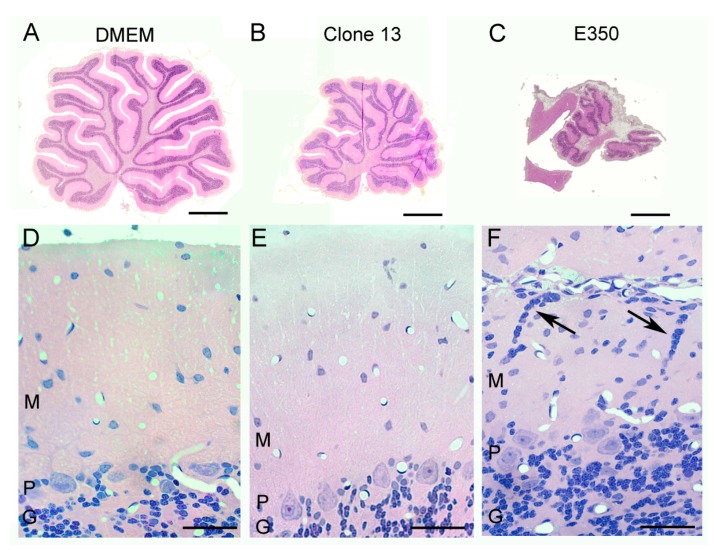
Strain E350, but not the other strains, induced a neuronal migration defect. Pictured here are 4-micron-thick, hematoxylin and eosin-stained, midsagittal cerebellar sections at low power (**A**–**C**) and high power (**D**–**F**) from rats on PD22. (**A**) The uninfected control cerebellum consists of ten lobules. (**D**) The normal cerebellar cortex consists of a cellular granule cell layer (**G**), Purkinje cell layer (*p*) in which large Purkinje cell bodies reside, and an outer molecular layer (**M**) in which cells are relatively sparse. (**B**) The Clone 13-infected cerebellum is markedly reduced in size (hypoplastic), but has no destructive lesions. (**E**) The cerebellar cortex of Clone-13-infected animals is histologically normal. (**C**) The E350-infected cerebellum is markedly abnormal, as it is greatly reduced in size and has large areas of encephalomalacia. (**F**) The cerebellar cortex of E350-infected animals is abnormal, with the presence of clusters of granule cell neurons (arrows) ectopically located within the molecular layer. These cells failed to migrate properly through the molecular layer to the internal granule cell layer. Scale bars represent 1 mm in **A**–**C** and 50 um in **D**–**F**.

These strain differences in Bergman glia pathology were reflected by strain differences in granule cell migration defects. Under normal circumstances, granule cells migrate from the external granule cell layer, through the molecular layer, to their adult location within the internal granule cell layer of the cerebellum. Normally, no granule cells remain within the molecular layer after PD 21. As shown in Figure 6, the control animals, which were injected with DMEM alone, have this normal configuration, with a large contingent of granule cells within the internal granule cell layer and none within the molecular layer. The Clone 13 animals, which had only minimal pathological changes in Bergman glia structure, also had the normal configuration. In contrast, the animals infected with the E350 strain, in which Bergman glia structures were substantially disrupted, had abnormal clusters of granule cells within the molecular layer (Figure 6). These ectopically located granule cells remain in their abnormal location due to failure of normal cellular migration, most likely due to the loss of the normal Bergman glia scaffolding.

### 3.5. Different LCMV Strains Had Different Cellular Targets of Infection

**Figure 7:**
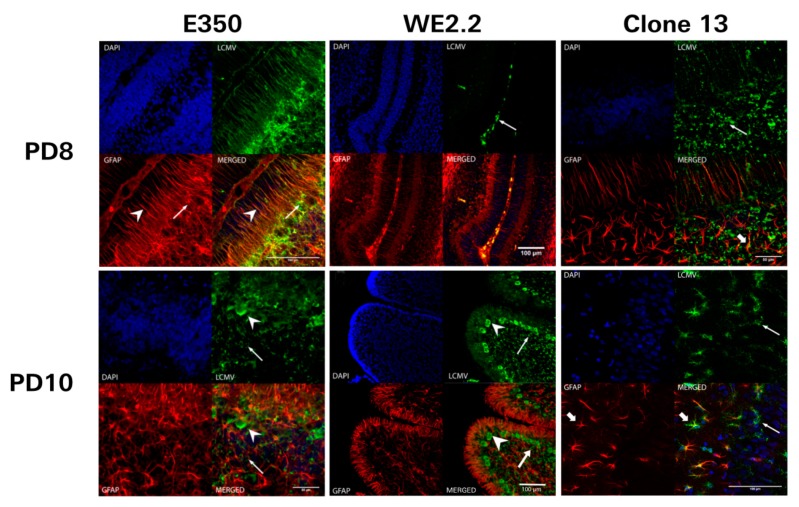
The different strains of LCMV had different initial cellular targets of infection and sequential migration. Pictured here are 40-micron-thick immunohistochemically stained sections (GFAP: red, LCMV: green, DAPI/nucleated cells: blue, bottom right: merged image) from PD8 (upper row) and PD10 (lower row) cerebellar tissue from rats infected with different strains of LCMV. For the animals inoculated with E350 (left column), Bergman glia (arrowhead) and astrocytes (arrows) are the initial cellular targets of infection on PD8. Thus, no neurons are infected by E350 on PD8, and all infected cells are GFAP-positive. However, by PD 10, E350 has spread to infect many granule neurons (arrow) and Purkinje neurons (arrowhead). This pattern of infection is in stark contrast to that observed with WE2.2 (middle column), where initial infection on PD8 is limited to the meninges (arrow) and no parenchymal cells are infected. By PD 10, WE2.2 has moved into neurons and exclusively infects granule neurons (arrow) and Purkinje neurons (arrowhead). In Clone 13, initial infection on PD8 was limited to granule cells (arrows). By PD10, the infection had spread to Purkinje cells (thin arrows) and a few astrocytes (thick arrow).

Because the rats of all three strains exhibited different degrees of cerebellar ataxia and pathology, the cerebellum was the brain region in which the cellular targets and sequential migration of infection for the three strains were compared and contrasted (Figure 7). For the E350 strain, Bergman glia and astrocytes were the initial targets of infection. Bergman glia and astrocytes are the cell populations within the cerebellum that express glial fibrillary acidic protein (GFAP). Following inoculation with E350 on PD 4, virtually all of the GFAP-expressing cells of the cerebellum were co-labeled with LCMV antibodies by PD 8. However, the E350 strain did not remain confined to these glial populations. By PD 10, the virus had spread into neuronal populations, including Purkinje cells and granule neurons, neither of which express GFAP. The virus was then cleared from Bergman glia and astrocytes so that, by PD 14, viral labeling was confined to Purkinje cells and granule cells and no longer present in glial populations. 

In marked contrast, Clone 13 did not initially target glial populations. Instead, for Clone 13, the initial targets of infection were granule cells, located within the external granule cell layer and internal granule cell layer of the cerebellum. On PD 8, four days following inoculation, LCMV-expressing cells were not co-labeled for GFAP. Over the course of time, Clone 13 spread from granule cells into Purkinje cells and into a few astrocytes. However, at no time did Clone 13 infect a large proportion astrocytes or Bergman glia, as was observed for E350.

The WE2.2 strain exhibited a completely different tempo and pattern of infection. On PD 8, a time point when the other strains showed clear parenchymal infection within the cerebellum, the WE2.2 strain showed no parenchymal infection. Instead, at PD 8, the WE2.2 infection was confined to cells within the meninges. The WE2.2 infection then rapidly spread to the cerebellar parenchyma. By PD 10, the virus was present within large proportions of cerebellar Purkinje cells and granule neurons. However, for the WE2.2 strain, viral antigens remained virtually absent from astrocytes and Bergman glia at all time points. Thus, the initial cellular targets of infection and the sequential migration of the virus varied considerably among the three LCMV strains.

### 3.6. Different LCMV Strains Elicited Different Host Immune Responses to Infection

Because brain injury in LCMV infections can be immune-mediated, and because the different strains induce different patterns and degrees of brain injury, the immune responses induced by the three strains of LCMV in the neonatal rat model were compared.

**Figure 8:**
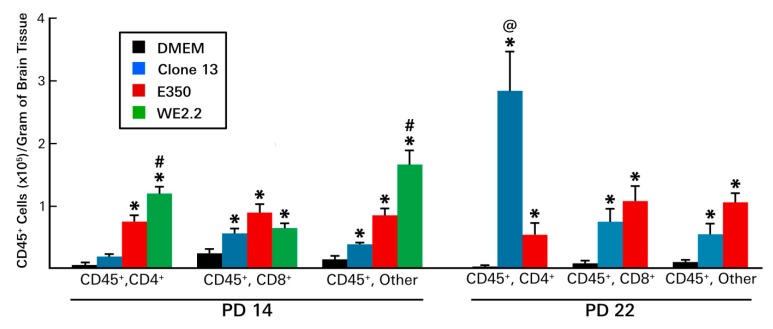
The different strains of LCMV induced different patterns of infiltrating immune cells. At PD14 (left panel): All three viral strains provoked infiltration of hemopietic cells. However, the infiltrations induced by the E350 and WE2.2 strains were much greater than that induced by the Clone 13 strain. In addition, among the strains, the identities of the infiltrating cells differed. For the E350 strain, CD8+ cells represented the plurality, while for the WE2.2 strain, only a small proportion were CD8+ and most were neither CD4+ nor CD8+. By PD22 (right panel), substantial changes in the profiles of infiltrating cells emerged. By this time point, Clone 13 had induced a large increase in the infiltration of CD4+ cells, a development not observed in the animals infected with E350. * = significantly different from DMEM control (*p* < 0.05); #=significantly different from all other groups (*p* < 0.05); @ = significantly different from all other groups (*p* < 0.01). Error bars represent standard error of the mean.

First, the quantity and composition of lymphocytic infiltrates were examined. As shown in Figure 8, large strain differences were observed in both the total number of lymphocytic cells and in their immunophenotypes. Furthermore, these differences between strains changed over the course of the infections.

At PD 14, lymphocytes could be isolated from all infected brains, and the number of those cells exceeded uninfected controls for all three strains. However, at PD 14, far more lymphocytes were present in the brains of the E350 and WE2.2 animals than in the brains of the Clone 13 animals. In fact, the number of lymphocytes in the E350 and WE2.2 animals exceeded that of the Clone 13 animals by more than two-fold and three-fold, respectively. Furthermore, among the strains, the infiltrating cells differed in their composition. For the E350 strain, the plurality of infiltrating cells were CD8+, while for the WE2.2 strain, only a small proportion were CD8+, and most expressed neither CD4 nor CD8 markers.

By PD 22, substantial differences in the lymphocytic profiles emerged, especially for the Clone 13 animals. Unlike PD14, when the Clone 13 animals had few infiltrating cells and the least cells among the strains, by PD22, the Clone 13 animals had a large number of infiltrating cells and the most among the strains. This large increase in the number of infiltrating cells in the Clone 13 animals was due almost entirely to an expansion of CD4+ cells. For the Clone 13 animals, while the CD4+ cells accounted for only a small proportion of the lymphocytic infiltrate on PD 14, they accounted for the great majority on PD 22. This expansion in the CD4+ population over time was strain-specific, as it did not occur in the E350 strain. The number of infiltrating lymphocytes for the WE2.2 animals could not be determined on PD 22, as none of the animals infected with that strain survived to PD 22.

**Figure 9:**
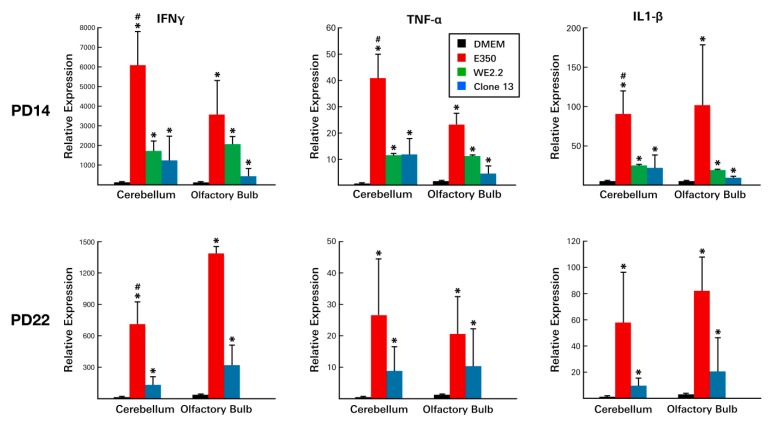
The different strains of LCMV induced different cytokine profiles. At PD14, all three viral strains provoked substantial increases in the expression of all three inflammatory cytokines. However, in both the cerebellum and the olfactory bulb, E350 provoked significantly greater cytokine responses than WE2.2 and Clone 13. At PD22, cytokine levels remained elevated, though not as high as at PD14. At PD22, E350 continued to induce substantially greater cytokine responses than Clone 13. * = Significantly different from DMEM control (*p* < 0.05); # = significantly different from all other groups (*p* < 0.05). Error bars represent standard error of the mean.

Next, the proinflammatory cytokine profiles were examined. Large strain-specific, brain region-specific, and time-specific differences in cytokine expression levels were observed (Figure 9).

On PD 8, significant elevations were observed in several cytokines within infected animals . However, these elevations were confined to certain LCMV strains and to certain brain regions. In particular, IFNg, IL-1B, and TNFa levels were increased as much as 100-fold. However, these increases occurred only in the E350 and WE2.2 strains and did not occur in Clone 13. Furthermore, the significant increases occurred only in the olfactory bulbs and were not observed in the cerebellum.

These same general relationships were observed on PD 14, except that substantial elevations were present not just in the olfactory bulbs, but also within the cerebellums (Figure 9). Furthermore, on PD 14, increased cytokine levels were especially prominent in the E350 infected animals, where several of the changes in cytokine levels dwarfed those seen in the other strains. By PD 22, cytokine levels had declined from previous peaks, but still remained elevated in the E350 and Clone 13-infected animals.

Thus, LCMV infection of the developing rat brain led to substantial changes in cytokine expression levels and to large lymphocytic infiltrations. However, the magnitude, time-course, and character of these immune responses varied considerably among the strains of LCMV. 

## 4. Discussion

This study utilized a neonatal rat model system of congenital LCMV infection to demonstrate that different strains of LCMV have profoundly different biological effects on developing animals in general, and on the developing brain, in particular. The different strains, several of which differ from each other by only a few nucleotides, produce different disease states, have different cellular targets of infection, induce different patterns of growth disturbance, lead to different forms of neuropathology, cause different behavioral deficits, and elicit different immune responses.

Clinical studies examining children with congenital LCMV infection have demonstrated that fetal exposure to LCMV can lead to widely different effects. In some cases, prenatal LCMV infection leads to fetal death, while others survive. In those who do survive, LCMV can cause a wide variety of neuropathological and developmental problems. The most common pathologic effects are chorioretinitis and microencephaly with periventricular calcifications [11]. However, others have very different findings, including porencephalic cysts, encephalomalacia, hydrocephalus, cerebellar hypoplasia, neuronal migration disturbances, and periventricular cysts. The neurodevelopmental outcomes of these children are also highly diverse. While some are profoundly cognitively impaired, others have only mild learning disturbances or vision loss, while still others are principally afflicted with ataxia, seizures, or cerebral palsy [10]. Because only symptomatic infants are likely to be tested, it is possible that congenital LCMV infection produces no adverse effects in some cases, and that the infection goes undetected. No epidemiologic studies investigating the rate of congenital LCMV have been conducted to investigate this possibility. 

This wide diversity in outcome among children with congenital LCMV infection suggests that the site, nature, and severity of pathology differ among cases. Why fetal infections with a single viral species would differ so much from case-to-case is unknown. However, one possibility is that they are infected with different *strains* of the virus. 

LCMV is an enveloped, bi-segmented, negative-strand RNA virus with an ambisense coding strategy. Each virion contains a long (L) segment RNA and a short (S) segment RNA. The L segment contains genes that encode for viral polymerase and a zinc finger motif protein, while the S segment contains genes encoding for the viral nucleoprotein and a glycoprotein precursor [39,40]. While all LCMV virions share this common structural arrangement, the nucleotides that constitute the RNA segments can vary, thus altering their genetic products. In fact, because of very high mutation rates, even cloned populations of LCMV and other RNA viruses do not consist of a single homogeneous species, but instead of heterogeneous mixtures of closely related genomes, which are referred to as “quasispecies.” [41]. LCMV nucleotide sequences diverge as much as 22% [6]. However, because these nucleotide differences do not lead to changes that can be easily detected serologically, morphologically, or with most standard molecular tests, LCMVs are classified as a single viral entity within the Arenavirus family [35].

Several dozen LCMV strains have been identified in nature and in the laboratory, and these strains can differ substantially in their biological properties. Experiments in vitro and in adult mice have shown that different strains of LCMV differ in their affinities for viral receptors, tissue tropisms, and courses of disease [19,34,42]. To our knowledge, only one study has examined the importance of LCMV strain in developing animals. Dutko and Oldstone [35] examined six strains of LCMV in neonatal mice and found substantial strain differences in lethality, liver disease, and persistence of infection.

Among children with congenital LCMV infection, the central nervous system is the main site of pathology. Yet, until now, no study has examined strain differences on brain pathology or dysfunction in an animal model of congenital LCMV infection.

We found that LCMV strains differ substantially in their biological effects on neonatal rats. Many of the differences that we observed among strains in the rats have a direct correlate to the differences observed among human babies with congenital LCMV infection and could explain some of the differences in outcome following prenatal infection with LCMV.

### 4.1. Different Strains of LCMV Differed in Their Lethality

While the animals infected with the WE2.2 strain universally died within several weeks of infection, almost all of the animals infected with the E350 strain survived. The animals infected with the Clone 13 strain had an intermediate survival rate. Differences in lethality are similarly observed in humans with congenital LCMV, in which the virus sometimes causes fetal demise, while other infected fetuses survive long-term [10]. While a multitude of factors, including viral load, gestational age, fetal pathology, and maternal and fetal immune responses likely all influence fetal survival in congenital LCMV infection, these results suggest that viral strain also plays an important role.

### 4.2. Different Strains of LCMV Differed in their Cellular Targets of Infection

For the E350 strain, the initial cellular targets of infection within the cerebellum were glial cells, including astrocytes and Bergman glia. Following infection of glia, the virus spread to neurons, including granule cells and Purkinje cells. This is precisely the same pattern of infection and sequential spread that has been observed with the Armstrong-4 strain, a strain to which E350 is closely related [27]. However, the pattern differs markedly from that of the Clone 13 and WE2.2 strains, in which neurons, rather than glia, were principally targeted. The strain differences in cellular targets may be due, at least in part, to differences in affinity for binding to viral receptors. Alpha-dystroglycan (aDG) is a known receptor for some strains of LCMV, but not others [43]. While Clone 13 has a high affinity for aDG, E350 and Arm-4 do not [19]. Affinity for aDG is not likely the whole explanation for the differences in infection patterns, however, since WE2.2 is similar to E350 and Arm-4 with a low affinity for aDG, yet differs from E350 and Arm-4 with a low propensity to infect glia.

The differential involvement of glial cells in LCMV infections may be critically important in the different neuro-immune responses to infection among the strains, since astrocytes are potent sources of cytokines and can interact strongly with other cellular components of the immune system. Indeed, the E350 and Armstrong-4 strains both prominently infect astrocytes and both induce robust immune-mediated destructive processes in the cerebellum and olfactory bulbs, none of which was observed with the other strains.

It is also notable that, several days following widespread infection of astrocytes and Bergman glia by the Armstrong-4 and E350 strains, viral antigen abruptly disappears from these glial populations and remains detectable only in neurons [27]. This clearance from glial cells may reflect the death of those cells or, more likely, the clearance of virus through a non-cytolytic process. Because the clearance from glial cells corresponds temporally with the initial infiltration of lymphocytes [27], we hypothesize that glial cells interact with lymphocytes to eliminate infectious virus. 

### 4.3. Different Strains of LCMV Differed in their Sites and Forms of Pathology

All three strains of LCMV interfered with brain growth. However, the spatial patterns of brain growth disturbance varied among the strains. E350 reduced brain weights, principally by inducing focal pathology specifically within the olfactory bulbs and cerebellum. Brain growth elsewhere appeared normal. In contrast, the Clone 13 and WE2.2 strains induced a generalized microencephaly in which all components of the brain were proportionally reduced in size. Even more striking were the markedly different forms of pathology among the strains. In particular, the E350 strain induced destructive processes that led to encephalomalacia and obliteration of large parts of the olfactory bulbs and cerebellum. In addition, the E350 strain induced neuronal migration disturbances, in which cerebellar granule cells remained ectopically located within the molecular layer. In stark contrast, the Clone 13 and WE2.2 strains had no destructive processes, but instead had hypoplasia of olfactory bulbs and cerebellum, and had no evidence of neuronal migration errors.

Previous studies have shown that, in rat pups infected with the Armstrong-4 strain of LCMV, the destructive lesions of the cerebellum are due to infiltrating CD8+ T-lymphocytes, while cerebellar hypoplasia is independent of those cells [28,30]. Thus, it is intriguing that, in the present study, cerebellar and olfactory bulb destruction occurred only in the animals infected with E350, which was also the strain that attracted the greatest number of infiltrating CD8+ T-lymphocytes, while hypoplasia occurred with all strains. These results suggest that, for all strains of LCMV, the destructive lesions are immune-mediated and driven by CD8+ cells, while hypoplastic effects are independent of T-lymphocytes. An important next step in this line of research will be to investigate the pathologic outcomes of infection with these viral strains in immunodeficient rats.

These strain-related differences in pathology have clear clinical correlates among children with congenital LCMV infection. Some prenatal infections with LCMV lead to destructive brain lesions, in which porencephalic cysts and regions of encephalomalacia are clearly evident. Others have no evidence of a destructive process, but have generalized microencephaly, cerebellar hypoplasia, or neuronal migration disturbances [10,11]. The results of this study suggest that these differences in neuropathology among LCMV-infected human fetuses may be due to infection with different strains of LCMV.

### 4.4. Strengths and Limitations of This Study

In this study, Lewis rat pups were uniformly inoculated with strains of LCMV on postnatal day 4. Thus, a major strength of this study lies in the fact that the infected animals were all genetically similar and infected at identical ages. As a result, differences in host genetics and age were eliminated as variables, and the large differences observed among the treatment groups must be attributed to differences in the viral strains themselves. Thus, the study’s results provide clear evidence that different strains of LCMV induce different diseases and neuropathology in a rat model of congenital LCMV infection. 

This study has several limitations. While the neonatal period in the rat closely resembles the second half of gestation in the human in terms of brain development, this is far less true with regard to the immune system [21,22]. Unlike the prenatal human, the neonatal rat is extrauterine and has no placenta or maternal immune system at work. Furthermore, the prenatal human and the neonatal rat are not highly comparable in the maturity of their immune systems. Thus, to the extent that some of the pathology is immune-mediated in congenital LCMV, the prenatal human and neonatal rat model may diverge widely. 

While this study demonstrates that infection with the different strains of LCMV induce substantially different forms and severity of neuropathology, the mechanism by which this occurs was not deeply explored. The different strains may have different replication kinetics, produce different viral loads in different brain regions, and produce different levels of defective interfering particles that differentially impact infectivity and pathology [44]. Our studies revealed that the infection with the different strains results in different patterns of lymphocytic infiltration and cytokine response, but the different strains may also have triggered different innate immune responses, which may have varied in different brain regions [45,46]. Furthermore, our studies revealed that the different strains differentially impaired spleen growth, but the mechanism underlying this growth failure and the contribution of spleen pathology to differential neuropathology remain unknown. All of these issues must be explored in future studies to understand the biological basis for the observations of this study.

All of the rats in this study received a uniform dose of infectious particles (1000 pfu) at a uniform age (PD 4) and were sacrificed for analysis during the preweaning stage. In one sense, this uniformity constitutes a strength of the study, as it eliminates potentially confounding variables of dose and host age. Indeed, we have previously shown that host age can critically affect infectivity and pathologic response in developing rats infected with LCMV [15]. However, effects of inoculation dose and age of infection may differ among the strains and may have important implications for congenital LCMV that were missed by exclusion of these variables from this study. In addition, because pathologic analyses in this study were confined to pre-weaning ages, differences in long-term outcomes induced by the different strains remain unknown. 

All three of the viral strains used in this study are well-adapted laboratory isolates of LCMV. The results demonstrate that different strains of LCMV can have substantially different effects in the developing brain. Thus, the results have translational value, as they support the notion that the different outcomes in congenital LCMV are due, at least in part, to differences in viral strains. Ideally, a next important step would be to repeat these studies with fresh isolates from congenitally infected human infants with different degrees of disease. Unfortunately, in most cases, LCMV has been cleared from the fetus by the time of birth and can no longer be isolated [8]. 

### 4.5. Neonatal Rat Infected with LCMV is a Powerful Model System of Human Congenital LCMV Infection

In a previous clinical study, we described the largest cohort to date of children with congenital LCMV infection [10]. We obtained clinical histories, physical examinations, and neuroradiologic studies, and we followed the children prospectively for 6 months to 11 years. In that cohort of congenital LCMV, we noted a broad spectrum of disease, especially in regards to the site, form, and severity of neuropathology. As a companion to that clinical investigation, we conducted a laboratory study in which we hypothesized that much of the variability in outcome among LCMV-infected fetuses is due to differences in the gestational timing of infection [15]. For that study, we infected experimental rat pups with the Armstrong-4 strain of LCMV and found that host age at the time of infection greatly affects patterns of infection, immune response, neuropathological effects, and disease course. Thus, gestational age is one key factor determining the effect of LCMV on the developing brain. The present study complements and greatly extends those previous results by showing that strain of LCMV is a second factor of central importance.

The neonatal rat infected with LCMV is a superb model system of human congenital LCMV infection. As shown in Table 1, the wide variety of disease outcomes and neuropathologic changes observed in humans with congenital LCMV can virtually all be replicated in the rat model by manipulating either the age of the rat at the time of infection or the LCMV strain to which it is exposed. Thus, because of the fidelity with which it reflects the human condition, the rat model of LCMV infection is probably the most powerful animal model of congenital infection in existence today [25].

## Figures and Tables

**Table 1:** Fidelity of the Neonatal Rat Model to Human Congenital LCMV Infection.

Effect in Humans	Comparable Effect in Rats	LCMV Strain	Infection Day
Fetal Demise	Early Death	WE 2.2	PD4
Microencephaly	Microencephaly	WE 2.2, Clone 13	PD4
Chorioretinitis	Chorioretinitis	Arm-4	PD1
Hydrocephalus	Hydrocephalus	Arm-4	PD10-PD60
Lissencephaly (Neuronal Migration Disturbance)	Neuronal Migration Disturbance	E350	PD4
		Arm-4	PD4-PD6
Porencephalic Cyst	Porencephalic Cyst (Olfactory Bulb)	Arm-4	PD10-PD60
		E350	PD4
Cerebellar Hypoplasia	Cerebellar Hypoplasia	Arm-4	PD1
		WE 2.2, Clone 13	PD4
Encephalomalacia	Encephalomalacia (Cerebellum)	Arm-4	PD4-PD6
		E350	PD4
	Encephalomalacia (Dentate Gyrus)	Arm-4	PD6
Periventricular Cyst	Periventricular Cyst (Occipital Horn)	Arm-4	PD10-PD60
Periventricular Calcification	Periventricular Infection	Arm-4	PD1-PD10

The data in this table was derived from a combination of the present study and several previous publications [15,25,27,47].

## References

[1] Stephensen C.B., Blount S.R., Lanford R.E., Holmes K.V., Montali R.J., Fleenor M.E., Shaw J.F. (1992). Prevalence of serum antibodies against lymphocytic choriomeningitis virus in selected populations from two U.S. cities. J. Med. Virol..

[2] Ambrosio A.M., Feuillade M.R., Gamboa G.S., Maiztegui J.I. (1994). Prevalence of lymphocytic choriomeningitis virus infection in a human population of Argentina. Am. J. Trop. Med. Hyg..

[3] Childs J.E., Glass G.E., Ksiazek T.G., Rossi C.A., Oro J.G.B., Leduc J.W. (1991). Human-rodent contact and infection with lymphocytic choriomeningitis and Seoul viruses in an inner-city population. Am. J. Trop. Med. Hyg..

[4] Fevola C., Kuivanen S., Smura T., Vaheri A., Kallio-Kokko H., Hauffe H.C., Vapalahti O., Jääskeläinen A.J. (2017). Seroprevalence of lymphocytic choriomeningitis virus and Ljungan virus virus in Finnish patients with suspected neurological infections. J. Med. Virol..

[5] Childs J.E., Glass G.E., Korch G.W., Ksiazek T.G., Leduc J.W. (1992). Lymphocytic choriomeningitis virus infection and house mouse (mus musculus) distribution in urban Baltimore. Am. J. Trop. Med. Hyg..

[6] Jahrling P.B., Peters C.J. (1992). Lymphocytic choriomeningitis virus: A neglected pathogen of man. Arch. Pathol. Lab. Med..

[7] Peters C.J. (2006). Lymphocytic choriomeningitis virus—An old enemy up to new tricks. N. Engl. J. Med..

[8] Bonthius D.J. (2012). Lymphocytic choriomeningitis virus: An under-recognized cause of neurologic disease in the fetus, child, and adult. Semin. Pediatric Neurol..

[9] Enders G., Varho-Göbel M., Löhler J., Terletskaia-Ladwig E., Eggers M. (1999). Congenital lymphocytic choriomeningitis virus infection: An underdiagnosed disease. Pediatric Infect. Dis. J..

[10] Bonthius D.J., Wright R., Tseng B., Barton L., Marco E., Karacay B., Larsen P.D. (2007). Congenital lymphocytic choriomeningitis virus infection: Spectrum of disease. Ann. Neurol..

[11] Wright R., Johnson D., Neumann M., Ksiazek T.G., Rollin P., Keech R.V., Bonthius D.J., Hitchon P., Grose C.F., Bell W.E. (1997). Congenital lymphocytic choriomeningitis virus syndrome: A disease that mimics congenital toxoplasmosis or cytomegalovirus infection. Pediatrics.

[12] Barton L.L., Peters C.J., Ksiazek T.G. (1995). Lymphocytic choriomeningitis virus: An unrecognized teratogenic pathogen. Emerg. Infect. Dis..

[13] Delaine M., Weingertner A.S., Nougairede A., Lepiller Q., Fafi-Kremer S., Favre R., Charrel R. (2017). Microcephaly caused by lymphocytic choriomeningitis virus. Emerg. Infect. Dis..

[14] Mets M.B., Barton L.L., Khan A.S., Ksiazek T.G. (2000). Lymphocytic choriomeningitis virus: An underdiagnosed cause of congenital chorioretinitis. Am. J. Ophthalmol..

[15] Bonthius D.J., Nichols B., Harb H., Mahoney J., Karacay B. (2007). Lymphocytic choriomeningitis virus infection of the developing brain: Critical role of host age. Ann. Neurol..

[16] Albariño C.G., Palacios G., Khristova M.L., Erickson B.R., Carroll S.A., Comer J.A., Hui J., Briese T., St George K., Ksiazek T.G. (2010). High diversity and ancient common ancestry of lymphocytic choriomeningitis virus. Emerg. Infect. Dis..

[17] Djavani M.M., Crasta O.R., Zapata J.C., Fei Z., Folkerts O., Sobral B., Swindells M., Bryant J., Davis H., Pauza C.D. (2007). Early blood profiles of virus infection in a monkey model for Lassa fever. J. Virol..

[18] Lukashevich I.S., Rodas J.D., Tikhonov I.I., Zapata J.C., Yang Y., Djavani M., Salvato M.S. (2004). LCMV-mediated hepatitis in rhesus macaques: WE but not ARM strain activates hepatocytes and induces liver regeneration. Arch. Virol..

[19] Smelt S.C., Borrow P., Kunz S., Cao W., Tishon A., Lewicki H., Campbell K.P., Oldstone M.B. (2001). Differences in affinity of binding of lymphocytic choriomeningitis virus strains to the cellular receptor alpha-dystroglycan correlate with viral tropism and disease kinetics. J. Virol..

[20] Dobbing J., Davis J.A., Dobbing J. (1981). The later development of the brain and its vulnerability. Scientific Foundations of Paediatrics.

[21] Dobbing J., Sands J. (1973). Quantitative growth and development of the human brain. Arch. Dis. Child..

[22] Dobbing J., Sands J. (1979). Comparative aspects of the brain growth spurt. Early Hum. Dev..

[23] Bonthius D.J., Winters Z., Karacay B., Bousquet S.L., Bonthius D.J. (2015). Importance of genetics in fetal alcohol effects: Null mutation of the nNOS gene worsens alcohol-induced cerebellar neuronal losses and behavioral deficits. Neurotoxicology.

[24] Goodlett C.R., Lundahl K.R. (1996). Temporal determinants of neonatal alcohol-induced cerebellar damage and motor performance deficits. Pharmacol. Biochem. Behav..

[25] Bonthius D.J., Perlman S. (2007). Congenital viral infections of the brain: Lessons learned from lymphocytic choriomeningitis virus in the neonatal rat. PLoS Pathog..

[26] Monjan A.A., Cole G.A., Nathanson N., Lehmann F. (1973). Pathogenesis of LCM disease in the rat. Lymphocytic Choriomeningitis Virus and Other Arenaviruses.

[27] Bonthius D.J., Mahoney J., Buchmeier M.J., Karacay B., Taggard D. (2002). Critical role for glial cells in the propagation and spread of lymphocytic choriomeningitis virus in the developing rat brain. J. Virol..

[28] Klein H., Rabe G.K., Karacay B., Bonthius D.J. (2016). T-cells underlie some, but not all, of the cerebellar pathology in a neonatal rat model of congenital lymphocytic choriomeningitis virus infection. J. Neuropathol. Exp. Neurol..

[29] Pearce B.D., Steffensen S.C., Paoletti A.D., Henriksen S.J., Buchmeier M.J. (1996). Persistent dentate granule cell hyperexcitability after neonatal infection with lymphocytic choriomeningitis virus. J. Neurosci..

[30] Monjan A.A., Cole G.A., Nathanson H. (1974). Pathogenesis of cerebellar hypoplasia produced by lymphocytic choriomeningitis virus infection of neonatal rats: Protective effect of immunosuppression with anti-lymphoid serum. Infect. Immun..

[31] Armstrong C., Lillie R.D. (1934). Experimental lymphocytic choriomeningitis of monkeys and mice produced by a virus encountered in studies of the 1933 St. Louis encephalitis epidemic. Public Health Rep..

[32] Bergthaler A., Flatz L., Hegazy A.N., Johnson S., Horvath E., Löhning M., Pinschewer D.D. (2010). Viral replicative capacity is the primary determinant of lymphocytic choriomeningitis virus persistence and immunosuppression. Proc. Natl. Acad. Sci. USA.

[33] Sevilla N., Kunz S., Holz A., Lewicki H., Homann D., Yamada H., Campbell K.P., de la Torre J.C., Oldstone M.B. (2000). Immunosuppression and resultant viral persistence by specific viral targeting of dendritic cells. J. Exp. Med..

[34] Kunz S., Sevilla N., Rojek J.M., Oldstone M.B.A. (2004). Use of alternative receptors different than a-dystroglycan by selected isolates of lymphocytic choriomeningitis virus. Virology.

[35] Dutko F.J., Oldstone M.B.A. (1983). Genomic and biological variation among commonly used lymphocytic choriomeningitis virus strains. J. Gen. Virol..

[36] Motz B.A., Alberts J.R. (2005). The validity and utility of geotaxis in young rodents. Neurotoxicol. Teratol..

[37] Smart J.L., Dobbing J. (1971). Vulnerability of the developing brain. II. Effects of early nutritional deprivation on reflex ontogeny and development of behavior in the rat. Brain Res..

[38] Carr W.J., Marasco E., Landauer M.R. (1979). Responses by rat pups to their own nest versus a strange conspecific nest. Physiol. Behav..

[39] Lehmann-Grube F. (1984). Portraits of viruses: Arenaviruses. Intervirology.

[40] Buchmeier M.J., Zajac A.J., Ahmed R., Chen I. (1999). Lymphocytic choriomeningitis virus. Persistent Viral Infections.

[41] Holland J.J.D., De La Torre J.C., Steinhauer D.A. (1992). RNA virus populations as quasispecies. Genetic Diversity of RNA Viruses.

[42] Ahmed R., Simon R.S., Matloubian M., Kolhekar S.R., Southern P.J., Freedman D.M. (1988). Genetic analysis of in vivo-selected viral variants causing chronic infection: Importance of mutation in the L RNA segment of lymphocytic choriomeningitis virus. J. Virol..

[43] Cao W., Henry M.D., Borrow P., Yamada H., Elder J.H., Ravkov E.V., Nichol S.T., Compans R.W., Campbell K.P., Oldstone M.B.A. (1998). Identification of alpha-dystroglycan as a receptor for lymphocytic choriomeningitis virus and lassa fever virus. Science.

[44] Welsh R.M., Lampert P.W., Oldstone M.B.A. (1977). Prevention of virus-induced cerebellar disease by defective-interfering lymphocytic choriomeningitis virus. J. Infect. Dis..

[45] Biron C.A., Nguyen K.B., Pien G.C. (2002). Innate immune responses to LCMV infections: Natural killer cells and cytokines. Curr. Top. Microbiol. Immunol..

[46] Suprunenko T., Hofer M.J. (2019). Complexities of type I interferon biology: Lessons learned from LCMV. Viruses.

[47] Monjan A.A., Silverstein A.M., Cole G.A. (1972). Lymphocytic choriomeningitis virus-induced retinopathy in newborn rats. Investig. Ophthalmol..

